# Recruitment of International Students Through a Synthesis of English as a Second Language Instruction, Social Justice, and Service Learning

**DOI:** 10.1007/s10755-020-09538-2

**Published:** 2021-01-23

**Authors:** Daisuke Akiba

**Affiliations:** Queens College & The Graduate Center, The City University of New York, Queens, NY USA

**Keywords:** Internationalization, International students, Japanese students, Regional institution, Service learning, Study abroad

## Abstract

Universities across the U.S. have increasingly emphasized internationalization, leading to rising numbers of international students attending U.S. institutions of higher education. However, these students tend to gravitate toward larger research-intensive universities with many other institutions seeing no increase in international student enrollments. Little is known concerning how to attract international students to regional institutions lacking name recognition. To address the above and promote internationalization through increasing the presence of students from abroad, an academic department at a regional public U.S. college used needs analysis to develop a pilot program for Japanese university students (*N* = 13). The program involved a synthesis of *English as a Second Language* instruction, *social justice* as a content area, and *service learning*, in a two-week credit-bearing summer session course. A post-participation survey revealed highly positive reactions, particularly in terms of working with local community members, and broad agreement that the program had been *life-altering.* The implications for international student program development at regional institutions are discussed.

## Introduction

Institutions of higher education^1^ have promoted the internationalization of their campuses in response to ever-evolving waves of globalization. There is an overarching expectation that increasing the presence of international students on campus affords all students with opportunities to refine their international competence and, consequently, participate more meaningfully in today’s interconnected world (Hudzik & Simon, 2012), making them more marketable in the global labor market (Kahn & Agnew, 2017). On a more institutional level, enrolling international students may bring financial benefits to universities through increasing the number of students paying higher out-of-state tuition at public institutions and compensating for declining domestic enrollment at many private institutions (Viggiano et al., 2018). International students are also likely to utilize auxiliary services such as student housing, further strengthening the revenue stream (Johnson et al., 2018). Finally, with internationalization being among the key parameters of institutional rankings, it is a way for colleges to gain institutional prestige (Cattaneo et al., 2016). Enrolling international students, thus, may bring a range of benefits beyond student learning.

However, for institutions without distinguished national or international name recognition, internationalization may be challenging (Hudzik & Simon, 2012). According to the International Institute of Education (IIE), 1,095,299 international students attended institutions of higher education in the U.S. in the 2018–2019 academic year, as both degree-seekers and non-degree candidates (e.g., those in English language programs), marking the highest number of international students in history (Institute of International Education, 2019). Furthermore, according to IIE data (2019), 55% of these international students attended institutions classified as Carnegie R1 Doctoral Universities: Very High Research Activities (Carnegie Classifications of Institutions of Higher Education, n.d.). Despite a continuous increase in international students over the last dozen years in the U.S., it seems that most international students enroll at a limited group of large, research-intensive institutions. Cantwell (2015) notes, not surprisingly, that the number of international students at master’s- and bachelor’s-level institutions has remained largely stable since 2000.

This imbalance primarily stems from certain institutions’ international presence and name recognition, typically associated with factors such as faculty research, branding, prestige, athletics, media exposure, desirable locations, and marketing efforts—all of which require substantial financial investment over long periods of time (Adams et al., 2012). Additionally, some universities have invested considerable resources into building global infrastructures, such as international branch campuses and overseas information centers, to achieve effective internationalization (e.g., New York University, 2020). However, with most American public universities facing continuous budget cuts in recent years and tuition revenues steadily declining at many private institutions (Johnson et al., 2018), frugality has become more prevalent in higher education. As a result, internationalization may currenty be less of an immediate funding priority at some institutions, as they juggle competing needs, such as teaching, learning, and research. Regional colleges and smaller universities typically have a local frame of reference, involving an institutional mission to serve the local community (Hudzik & Simon, 2012), and this may put internationalization outside of their top funding priorities. Given this backdrop, Hudzik (2015) postulates that not all institutions should try to compete head-to-head with large Carnegie R1 institutions in recruiting international students, but rather pursue an alternative niche market. Perhaps reflecting the limited market share held by smaller, often regional institutions, there has been little research on how such institutions can optimize their international recruitment efforts (James-MacEachern & Yun, 2017).

### Navigating the Uneven Playing Field

What should universities without wide name recognition or robust resources do to attract international students and host programs—that will be beneficial to both the participating students and the host institution—within the constraints discussed thus far? An increasing number of institutions have sought services provided by third-party proprietary vendors that specialize in student recruitment (Farakish et al., 2020; Redden 2018a). However, Nikula & Kivistö (2020) warn that there are no transparent or objective data confirming these vendors’ ability to successfully boost international enrollment. Additionally, while Redden (2018b) describes a few cases in which the target for international enrollment was modestly or substantially met, she also reports a number of less-than-successful partnerships, including a case in which a state university reported a significant financial loss and a decline in the admissions standards, as a result of entering into partnership with a third-party vendor, putting the sustainability of these partnerships into question. While a detailed discussion of these oft-controversial third-party recruiters is beyond the scope of this study, it is relevant to note here that each of these vendors has a large portfolio of partner universities, including global elites that already dominate the market and may be better able to afford higher commissions (Redden, 2018a). Given that these proprietary vendors are driven by profit rather than equitable distributions of international students among institutions, it would make strategic sense for non-global elites to explore additional methods of internationalization.

Abundant studies have examined factors contributing to international students’ choice of institutions in general. By contrast, however, little is known concerning how regional institutions without global recognition could appeal to prospective international students, according to James-MacEachern (2018), who has been a leader in voicing concerns over the lack of literature regarding such regional, and often smaller, institutions. This dearth of literature is curious because scholars have previously suggested that “underdog” institutions with more regional foci develop their own unique strategies to pursue internationalization, including non-degree programs and programs that offer unique experiences outside of the classroom (Hudzik & Simon, 2012). While anecdotal success stories among smaller liberal arts colleges have been reported, such as Beloit College in Wisconsin (Brewer, 2015), no systematic understanding of how best to foster internationalization at regional institutions has been developed*.*

 James-MacEachern and Yun (2017) surveyed 242 international students at University of Prince Edward Island (UPEI), a regional public university in Canada. Contrary to previous research pertaining largely to research-intensive institutions, ranking was not found to have been the primary determinant of the destination institution choice among this population. Instead, their results pointed to the importance of a match between the varying needs of prospective international student populations and what the institution offers. For instance, while UPEI students from China tended to prioritize post-graduation employment opportunities which will permit them to remain in Canada, their counterparts from other countries emphasized financial affordability. James-MacEachern (2018) thus suggests that regional universities should be particularly sensitive to “consumer” needs when designing international programs for specific target populations. This observation, combined with Miller et al.,’s (2015) assertion that institutions can enhance their international appeal through designing programs with distinctive features, highlights the critical importance of exploring the needs of prospective students, which, in turn, reinforces the suggestion by Hudzik and Simon (2012) that regional institutions need to explore their own marketing niche in order to internationalize effectively. In response to these considerations, a pilot program for international students was developed and delivered at a regional U.S. institution, as described and analyzed below.

### Service Learning for ESL Students

This pilot international program utilized service learning (SL). Ehrlich (1996) defines SL as mutually enhancing academic learning and real-life experiences, following an educational philosophy advocated by John Dewey that considers academic learning and experiential components as inseparable in optimizing authentic learning (Dewey, 1938). Additionally, SL takes place in credit-bearing courses that create synergetic effects between academic content and service experience to promote both academic learning and citizenship (Bringle & Hatcher, 1995).

SL has been extensively and successfully implemented in North American universities (Askildson et al., 2013), and it has also been common for U.S. students to go overseas to participate in SL as part of their study abroad experiences (Crabtree, 1998). In addition to the overall value SL brings to college learning, several researchers have emphasized how SL in English as a Second Language (ESL) contexts can promote:

- Language development (Heuser, 1999; Marlow, 2007; Minor, 2001; Spack, 2006; Wurr, 2009);
- Linguistic self-confidence and general academic performance (Hummel, 2013); and
- Social awareness (Askildson et al., 2013; Perren et al., 2013).

The benefits of incorporating SL into language instruction for ESL students have been widely discussed for over two decades. However, few programs have put this idea into practice, and, in fact, when SL is integrated into ESL programs, international students typically find themselves receiving rather than providing service (Chao et al., 2017; Grassi et al., 2004; Sun & Yang, 2015).

Northeastern University has developed an internship-based international pathway program designed to help ESL students gain fluency in English through fieldwork where they take on various roles, ranging from receptionists at assisted-living facilities to assistants in afterschool programs (Miller et al., 2015). Bunning and Kostka (2018) had participants in this program reflect on their experiences and concluded that fieldwork in which students were compelled to communicate in English effectively advanced their English learning. This is consistent with previous research that attributes SL’s effectiveness in ESL instruction to encouraging a mindset whereby English language acquisition is considered more in terms of a social tool rather than as a goal in its own right (Grabois, 2007). However, Northeastern University’s program, focusing on acquisition of language skills through fieldwork, does not emphasize content-area academic knowledge, which may divert from the spirit of SL as content-based inquiry (Bringle & Hatcher, 1995).

### The Current Conceptual Framework

**Fig. 1:**
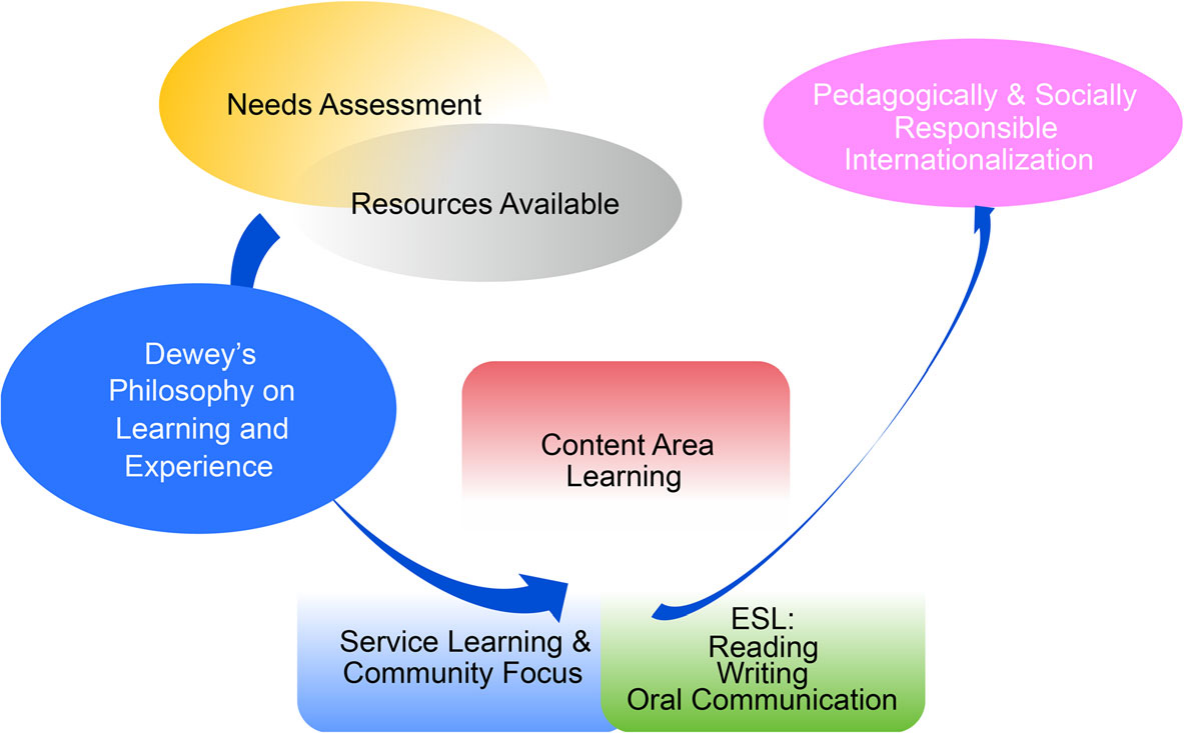
A conceptual framework, informed by John Dewey’s vision concerning learning and service

After considering the relevant literature in developing a cohesive and effective approach, the pilot program sought to: (a) align with the needs assessment of targeted international students while taking advantage of what the institution had to offer (Hudzik, 2015; James-MacEachern, 2018); (b) infuse ESL instruction with content-area academic instruction through SL while remaining faithful to the definition of SL (Bringle & Hatcher, 1995); and (c) emphasize pedagogically sound and socially responsible practices in accordance with the institution’s commitment to the local community (Hudzik & Simon, 2012). This approach accorded with Dewey’s (1938) philosophy, in which optimizing learning through drawing direct connections between classroom learning and corresponding participatory experiences is central to pedagogical conceptualization. Within this pilot ESL program, greater emphasis was put on ESL instruction conceived as a tool of communication rather than as a standalone learning goal, following Grabois’s (2007) approach. Specifically, ESL was envisaged as a means to integrate content-area learning and fieldwork experience. Figure 1 presents the conceptual framework of the pilot program.

## Method

### The Pilot Site

Prince College (pseudonym) is a regional public institution of higher education in the American Northeast, with a Carnegie Classification of Master’s Colleges and Universities. It is not ranked in major ranking systems nationally or internationally, although it consistently ranks among the top 10 in the regional *Top Public Schools* category of *U.S. News and World Report’s Best Colleges* (U.S. News & World Report, 2020). Approximately 15,000 undergraduate students are enrolled, and its domestic student body is highly diverse, with an equal split among Asian, Latinx, and White students (approximately 30% each), while Black students make up less than 10%. Recently, Prince College has enrolled approximately 500 to 650 international students annually, as part of a college-wide drive to promote internationalization.

Located about an hour on public transportation from the center of a large city, one of the college’s core missions is to serve the local community. Although it is primarily a commuter institution, there is an on-campus dormitory. International undergraduate students pay approximately US$19,000 in tuition and fees annually (approximately US$890/credit in summer), which is markedly lower than the cost of local private institutions where, at a similar-sized neighboring private institution, the annual tuition is around US$45,000.

The development of the pilot program was led by an academic department that almost exclusively specializes in certification-bearing teacher preparation. Probably due to the heavy focus on teacher certification, international students do not routinely enroll in this department. The pilot project was undertaken to stimulate international enrollment within this department as well as to contribute to college-level internationalization initiatives, by creating a program that is designed to cater to the interests and needs of the university students in Japan.

### Phase 1: Needs Assessment

Because the department chair spearheading the project was scheduled to visit Japan on two separate occasions for conferences, seminars, and research meetings in 2018, Japanese students were selected as the target population for this pilot program. Subsequently, *needs assessment* was conducted through conversations with undergraduate students from universities across Japan who were attending these gatherings. These conversations, which were made possible by assistance from the organizers of these events, were structured as both individual interviews and small group discussions of fewer than 10 students. During these conversations, Japanese university students were asked open-ended questions about their views on: (a) study abroad opportunities in general; (b) destination preferences; (c) program duration; (d) the destination institution’s ranking or name recognition; (e) learning goals; (f) program structure; and (g) cost. Additionally, the department chair was able to call on the assistance of colleagues from Japan’s Honda University (pseudonym), who were instrumental in facilitating additional conversations with students at this university. In total, approximately 40 students provided feedback.^2^ These discussions revealed five major areas of needs, as outlined in the left column of Table 1.

**Table 1:** The needs identified and corresponding resources available at Prince College

Needs identified	Corresponding resources and features at Prince College
Students prefer to attend short-term, non-degree ESL programs (two weeks to one semester in duration).	Prince College offers short-term, non-degree options for international students.
Destination institution’s ranking would matter if obtaining degrees. For non-degree study abroad, rankings are not an important consideration.	Prince College’s ranking is perhaps a non-issue for short-term, non-degree studies.
Improving English and interacting with locals are two major goals in going abroad.	Prince College offers quality ESL instruction, and its SL programs provide meaningful opportunities to interact with locals.
Rather than learning English through traditional methods, students would like to take content-area courses, ideally for academic credit, through which to improve their English.	One of the College’s SL programs could provide the context in which to develop a credit-bearing, content area-based ESL program.
Cost is likely to be the final determinant in choosing destination institutions.	Prince College’s lower tuition is advantageous.

In summary, it was revealed that: (i) Japanese university students were more eager to attend short-term English language courses overseas than long-term degree programs, presumably because diverting from their prescribed course of study at home institutions could complicate their future career prospects; (ii) Prince College, with its strong regional presence and without global name recognition, appeared suitable for short-term programs among this population; (iii) students looked to connect with the local community; (iv) they valued a content area-based ESL program that was ideally credit-bearing; and (v) cost was an important factor. Remarkably, these needs, aside from cost, had not been emphasized in the literature reviewed, which further corroborates the importance of regional institutions undertaking thorough due diligence when considering the needs of the potential “target audience” in designing international programs (Hudzik, 2015; James-MacEachern, 2018).

In early fall of 2018, the department chair met with various Prince College stakeholders, such as the department faculty, the Study Abroad Office, and the college’s upper administrators, in order to assess how well these identified needs could be matched with the college’s resources. The right column of Table 1 assesses how Prince College was able to meet each of these needs. The program was then designed based on that assessment.

### Phase 2: Program Design and Logistics

The host department at Prince College was confident that it could respond effectively to all of the needs identified during Phase 1, as seen on the right column of Table 1, within its available resources. Notably, it has a long-standing relationship with homeless family shelters through its community service programs, whereby students visit the shelters regularly and provide mentorship to the resident children. Youth homelessness has become a major community concern in the region in which the college is located (Shapiro, 2019) and, in relation to a social justice framework, this topic was assessed as practicable for developing an SL program. Youth homelessness is a significant social concern and worthy of rigorous scholarly inquiry, and SL based on this topic would permit students not only to serve the local community but also to deepen their understanding of social justice in real-life context, as well as to develop their English fluency. Specifically, the program was designed to improve students’ English language fluency and knowledge of social justice while serving the local community in relation to four pedagogical pillars: (i) reading and writing assignments on social justice with a special attention paid to youth poverty; (ii) oral discussions and written activities in class linking social justice with fieldwork; (iii) guided activities at a local homeless shelter; and (iv) post-visit reflections utilizing oral and written communication. With these considerations in mind, the blueprint for the pilot program was drawn up as outlined in Table 2.

**Table 2:** Basic construction of the program and the underlying rationale

Program features	Rationale
A two-week intensive summer session program	• To serve the demand for a short-term program
	• To curtail the overall cost of stay
	• To gain enrollment during the period when campus facilities typically remain underutilized
A credit-bearing service-learning seminar on social justice for ESL students (two semester credits)	• To acknowledge a rigorous academic component beyond ESL, worthy of two credits
	• To facilitate community involvement
	• To enhance ESL learning
A *Social Foundations of Education* lecturer appointed to teach in the program	• The instructor has experience in both social justice and ESL
Enrollment was restricted to students from Honda University	• To ensure smooth operation for the inaugural cohort (e.g., students with similar academic backgrounds)

The two-week course, delivered over 10 calendar days, included 3 hours of class time each morning of which 3 days were to be dedicated to on-site activities at a local homeless shelter. Because the fieldwork site, a homeless family shelter, had an existing relationship with the host academic department, no particular arrangements were required apart from notifying the shelter that the student visitors comprised English learners from Japan. The shelter did not raise any concerns; on the contrary, the staff welcomed the opportunity for residents to interact with people from backgrounds they would not ordinarily encounter. During this phase, the host department began announcing the preview of the program on its website and through colleagues at Japanese universities, with additional plans to publicize it through such not-for-profit advocates as *JAFSA: Japan Network for International Education*. Honda University, however, requested that the program enrollment be limited to its students. Since the host department was agreeable to the idea of keeping the inaugural cohort small, other participants were not recruited in the end.

In terms of cost to participants, the out-of-state summer tuition and fees for this two-credit course were approximately US$1,900, including books, materials, and fieldwork supervision. In addition, students paid US$65/night for campus housing and were responsible for other expenses, such as transportation, meals, and incidentals. From the departmental budgetary standpoint, the instructor and a half-time teaching assistant were paid according to the regular summer session salary rate, with no additional administrative costs incurred.

### Phase 3: Program Implementation

Thirteen (10 females and 3 males) Honda University students participated in this undergraduate-credit-bearing program in summer session 2019. Honda University coordinated the trip from Japan to Prince College as well as the rest of the pre-arrival details, such as student visa clearance and insurance. While not a requirement, Honda University opted to send and pay for a chaperone, who was the director of Honda’s School of Education and Human Development’s International Office.

The department chair communicated with the chaperone to address questions and concerns throughout the program. Here, two issues are noteworthy. First, the instructor did not consistently start the class precisely on the dot, frustrating the students from a highly punctual culture (Okazaki, 2012). While delays by a few minutes might not necessarily seem like a critical concern, the department made a good-faith effort to resolve this matter to ensure punctuality and supplement the missed class time. Second, some students expressed concerns about the discussion-based seminar. Reportedly, at their home institution, even small seminars are taught as lectures, and discussions are presumably considered less academically rigorous than lectures. The chaperone consequently asked that consideration be given to converting the discussion-based seminar to a lecture-based one.

The hosting department called an emergency meeting to discuss this issue at length, consulting with various faculty experts; subsequently, it concluded that it would not be pedagogically sound to divert from the seminar format in an integrated SL course with an explicit emphasis on helping students improve English. Consistent with Grabois’s (2007) view, the faculty unanimously agreed that discussions provided a vital means to oblige ESL students to actively communicate in English and, equally importantly, be exposed to the interactive teaching styles prevalent in the U.S. The department chose to keep the seminar format and asked the instructor to make minor modifications, such as breaking down discussion questions into smaller, more manageable components and providing frequent feedback, to accommodate further the students’ linguistic limitations. In the end, all 13 students passed the course successfully.^3^ These cultural nuances, beyond what is apparent from the research literature, exemplified the expectations some international students may bring with them that may affect their learning experiences. Therefore, feedback from the students participating in the pilot program, collected through a post-participation survey, needs to be carefully considered in planning for future cohorts of the program.

## Post-Participation Survey Findings and Discussion

Program participants were asked to complete an anonymous online post-participation survey in Japanese, conducted by the hosting department. Of the 13 students who participated in the program, 11 (84% of the students participating in the pilot program; nine females) completed the survey. The Institutional Review Board granted an exemption for this study on the basis that it consists of an analysis of existing data from an anonymous student survey, which had been administered as part of program evaluation. The survey included questions regarding program satisfaction, feedback on structure and content, and program impact. The survey included Likert-type questions, some of which were followed up with open-ended questions asking the participants to elaborate on their answers. Given the small sample size, no inferential statistics were performed. While the results are discussed in detail below, Table 3 lists the mean and standard deviation for each of the key Likert-type questions.

**Table 3:** Post-participation ratings provided by project participants

Statements	Means & standard deviations (SDs)
1: “Completely Agree”; 5: “Completely Disagree”	
I am satisfied with the program, overall.^a^	1.11 (.33)
I am satisfied with the service- learning component of the course. ^a^	1.25 (.45)
I am satisfied with the program format integrating social justice, service learning and the English language. ^a^	1.18 (.37)
I am more comfortable interacting with people from different cultural backgrounds.	1.82 (1.05)
Participating in this program made me want to learn English further.	1.45 (.43)
Participating in this program made me consider participating in a longer-term program.	2.44 (1.01)
My command of English has improved.	2.36 (1.00)
I had great interactions with locals.	1.81 (1.32)
This program was positively life-altering. ^a^	1.09 (.33)

^a^Open-ended follow-up responses were obtained on these questions

### Overall Satisfaction

On a scale from 1 (completely satisfied) to 5 (not at all satisfied), the participants expressed substantial satisfaction with the program. Among the nine responses obtained on the follow-up open-ended questions probing the program’s overall strengths,^4^ two themes clearly emerged: (i) course content and (ii) working with the local community. Specifically, five answers (45%) were themed around “course content and delivery” (e.g., firsthand experience with families in economic and housing distress, or learning about how social justice may play out in real-life settings), while four answers (36%) cited interaction with local children as the program’s strength. Directly or indirectly, all these responses pertain to working with the local community, which is consistent with Mahmood and Burke’s (2018) finding that community participation and international student satisfaction were linked. The remaining answers pinpointed campus aesthetics and low cost (*n*=1, or 9% each). Incidentally, with the exception of campus aesthetics, all the answers were directly relevant to the dimensions identified during the needs assessment process, signaling that the needs analysis was effective.

When asked about areas of improvement overall, four (36%) expressed disappointment that they did not have much interaction with Prince College students. The remainder of the answers included one (9%) each concerning the following: (a) a desire for more organized activities, such as parties and fieldtrips; (b) feeling uneasy about speaking up in class; (c) neighborhood safety concerns; and (d) the total cost being higher than anticipated. Since this program took place during the summer session, there were much fewer people, events, and activities on campus than there ordinarily are during the academic year, which may have also contributed to safety-related concerns. In light of these responses, forums whereby international and domestic students interact are to be organized when running future programs.

### The Service Learning (SL) Component

The survey participants indicated overwhelming satisfaction with the SL component. When asked to comment on the program’s strengths open-endedly in terms of SL, two major themes around community involvement emerged. First, four responses (36%) noted interaction with children had been a strength, with two participants specifically explaining that children made ideal conversational partners because of their open-mindedness. As one participant commented, *“Children are so curious and nonjudgmental that they are happy to talk to you, even if you cannot communicate smoothly in English.”* Second, three answers (27%) alluded to being able to take an inside look at the local community as a strength. One remaining answer (9%) noted that, through SL, the participant had overcome a fear of the unknown.

The notion of children being ideal conversational partners is particularly noteworthy. On a related note, besides evidence of its pedagogical effectiveness, research has demonstrated that pairing ESL students with elderly volunteers can be personally gratifying to both students and volunteers (Lai and Kaplan, 2016; Schnack, 2004). A similar possibility appeared to operate here between international students and children, at least from the students’ perspective, which presents an interesting topic for future research on ESL instruction. If such an arrangement is shown to benefit both parties, intergenerational interactions between international students and local children may provide additional layers of options when designing effective international programs.

At the same time, some concerns were raised about the SL component. Four responses (36%) noted occasional challenges in interacting with children, with two suggesting that the pre-fieldwork seminar could have included more concrete tips on managing children’s behavior (e.g., how best to handle children when they are too energetic). These responses provide guidance for future planning, for example, that more attention needs to be paid to supporting students in terms of “behavioral management” in fieldwork.

### The Synthesized Seminar Format

The survey participants expressed strong satisfaction with the class, which had sought to seamlessly synthesize ESL and social justice through an application of SL. When asked to elaborate their reasons, two themes emerged. Three participants (27%) commented on how useful it was to be obliged to speak English throughout the day. Two additional answers (18%) indicated that learning English as a tool, rather than studying English as an end in itself, made the experience more positive. These responses directly corroborate studies by Grabois (2007) and Bunning and Kostka (2018). The respondents seem highly satisfied with the format in which ESL instruction was framed as a means to accomplishing other ends, such as content-area learning and community service. Concerning the seminar format, only one area for improvement (9%) was noted, namely, that participants be given options from a list of content-areas to choose form. However, while offering a range of other topics is a useful future consideration, implementing this suggestion would require resources greater than the host institution currently has to offer.

### Program Impact

In terms of responses to the Likert-type questions, moderately positive ratings were obtained on two questions: improved fluency in English and increased interest in pursuing a longer-term study abroad in the future. These responses were not unexpected, given the short-term nature of the pilot program, with a likely limited effect on one’s linguistic skills or on attitudes toward a long-term study abroad.

The participants gave more positive ratings for the other questions, in terms of an increased interest in studying English further, having had positive interactions with locals, and becoming more comfortable interacting with people from other cultures. The most notable result was obtained concerning the item: *This program was positively life-altering*. The clear agreement among the participants that the program had had an exceptionally positive impact on their lives, as indicated by the small standard deviation of .33, was especially gratifying for the program organizers. Open-endedly, all seven responses obtained (63%) concerned self-discovery and personal growth. Of these, three responses specifically alluded to the realization of their privileged upbringing. One participant stated, “[Participating in the program and interacting directly with financially-distressed families experiencing housing instability]…*made me realize how lucky I have been. All the hardships I thought I had faced in my life, in the bigger scheme of things, weren’t hardships at all.*” These responses provide strong evidence that the participants left the program with personal accomplishments far greater than language acquisition or academic learning. Specifically, these students seem to have gained life-altering experiences through personally tackling a specific social issue involving children and their families.

## Conclusions and Limitations

The pilot program comprised three notable characteristics. First, informed by previous research, this project was developed using a needs analysis of the target population. The post-participation survey revealed that the features identified in the needs analysis were overwhelmingly noted as positive features of the program, suggesting that the needs analysis was effective. This step was vital to the success of the program, given the dearth of literature on “best practices” for institutions without major name recognition to attract international students. As highlighted earlier, a number of specific needs, not explicitly identified in previous research, were discovered through this process. Prince College’s modest international name recognition was used as an opportunity to design a short-term SL program with a social justice focus, which is unusual for an ESL program, that capitalized on the institution’s existing relationship with the local community. This led to the development of a program that is pedagogically and socially responsible, resource-conserving, and, perhaps most importantly, satisfying to the participating students.

Second, this credit-bearing program was spearheaded by an academic department, in coordination with various stakeholders. Compared to more formalized programs offered by universities, this approach gave the program greater control and flexibility. For example, when a concern was raised about seminar discussions, the department was able to make changes to address the issue swiftly and amicably. From a budgetary standpoint, since the academic department conducted the summer program at no administrative cost to the institution, the program provided an example of a promising non-resource-intensive method for internationalization. Although the number of participating students was just average for a summer class, they paid out-of-state tuition—set at roughly three times higher than in-state degree-candidate summer tuition, and they all fully utilized auxiliary campus services (e.g., campus housing) that often remain grossly underutilized over the summer. While the program thus generated notably higher earnings for the college than a typical summer class, students did not pay beyond the cost of taking a regular summer session course; naturally, the academic credits gained may be transferred to other universities to the extent that those institutions allow.

Third, following Dewey’s (1938) educational philosophy and from a curricular perspective, this pilot program represented a holistic integration of *social justice education* and *SL* with an underlying focus on *ESL*, designed to create a synergistic effect to enhance student learning in an authentic manner while emphasizing the college’s mission to serve the local community. Findings from the post-participation survey revealed overwhelming student satisfaction with this pedagogical approach and showcased a program providing a life-altering experience, in addition to language learning.

There are some limitations to be considered. First, the department chair received occasional assistance from his colleagues in Japan, which is an advantage others may not necessarily enjoy. Furthermore, since the pilot participants were restricted to Honda University students, student recruitment efforts were minimal. Still, based on the volume of inquiries the host department received in response to the initial announcement released on its website, as well as through colleagues at Japanese universities as described earlier, it speculates that a greater number of students could have participated, had it opted to recruit others. Expanding the program, nevertheless, needs to be approached with caution, as forming a small and cohesive cohort may have contributed to participant satisfaction. Finally, the outcomes were measured through survey-based self-reports without other sources of assessment, and the sample size was small at 11 survey participants. Future program assessment data should include other measures, such as academic and linguistic achievement benchmarks, to afford a more comprehensive program evaluation.

Circumstances related to the COVID-19 pandemic have made international travel impractical, and the future of internationalization in higher education remains uncertain. Nevertheless, when conversations resume concerning future cohorts for the program, close attention will be paid to the pilot student feedback to ensure that the positive features identified are enhanced while areas for improvement are addressed as appropriate. Overall, this pilot program offers a helpful approach in designing and hosting international student programs more effectively, where institutional features, priorities, and resources are used to their full advantage. It would be of critical importance for other institutions seeking to apply a similar program to closely scrutinize the needs of the potential target population in the context of their institution’s strengths, weaknesses, institutional priorities, and available resources. When this process is applied effectively in developing a cohesive and coherent strategy, the resulting international student program is more likely to be successful.

### Availability of Data and Material

Not applicable.

## Footnotes

1 In this manuscript, institutions of higher education, universities, and colleges are used interchangeably.

2 The exact count of students consulted was not recorded since, initially, these conversations were held for pilot program development rather than for academic research.

3 Access to detailed course grades was not granted by the Institutional Review Board, which noted that the small sample numbers would make maintaining confidentiality and anonymity difficult, even with the use of institutional pseudonyms.

4 In these follow-up open-ended questions, participants were asked to name notable positive features and areas of improvement, following each Likert-type question.
